# Renal replacement lipomatosis: A rare type of renal pseudotumor

**DOI:** 10.4103/0971-4065.65303

**Published:** 2010-04

**Authors:** N. A. Choh, M Jehangir, S. A. Choh

**Affiliations:** Department of Radiodiagnosis, SMHS Hospital, Soura, India; 1 Department of Pediatrics, Sher-i-Kashmir of Institute of Medical Sciences, Soura, India

**Keywords:** Pseudotumor, lipomatosis, kidney

## Abstract

Replacement lipomatosis of the kidney is the end result of severe atrophy of renal parenchyma with secondary marked proliferation of renal sinus and perirenal fatty tissue. Although ultrasonography may suggest the diagnosis, CT demonstrated the distinctive features most accurately. We report a case of renal replacement lipomatosis with coexistent xanthogranulomatous pyelonephritis and multiple perinephric and parietal wall collections.

## Introduction

Renal replacement lipomatosis (RRL) is a rare and benign condition characterized by proliferation of renal sinus/hilar and perirenal fatty tissue with marked atrophy of the renal parenchyma. It is seen with calculus disease in 70% of cases and associated with chronic inflammation and hydronephrosis.[1] This entity may mimic and can be confused with a fatty neoplasm of kidney. We report CT features of a case of RRL and coexistent xanthogranulomatous pyelonephritis in an elderly male, with emphasis on the differential considerations.

## Case Report

**Figure 1:**
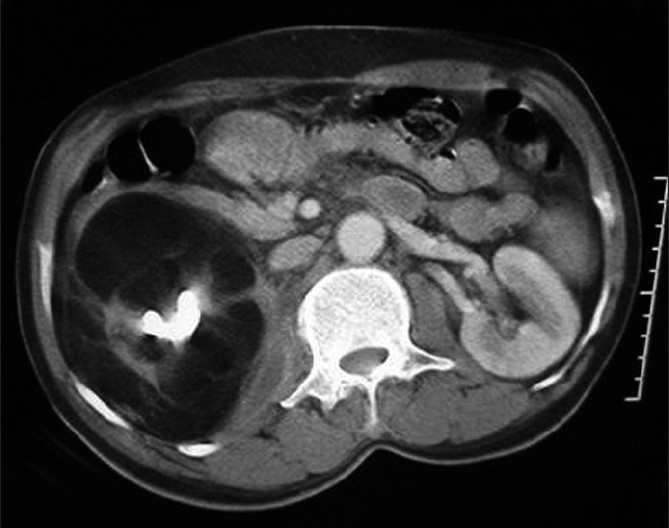
Contrast-enhanced CT of abdomen shows replacement of the right kidney by fat with a large central hyperdense calculus. A small posterior perinephric collection is noted extending into the adjacent psoas. Thin residual strands of renal tissue are noted

**Figure 2:**
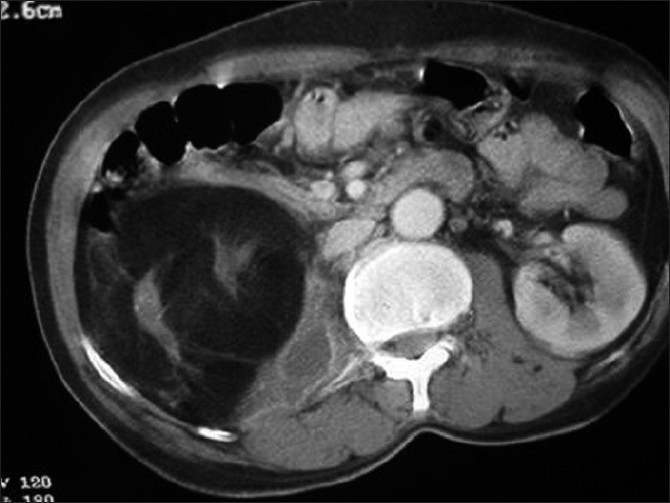
Contrast-enhanced CT of abdomen shows replacement of right kidney by fat with a large central hyperdense calculus. A small posterior perinephric collection is noted extending into the adjacent psoas. Thin residual strands of renal tissue are noted

A 60-year-old man presented with a subacute history of right flank pain and low-grade fever. His physical examination revealed an ill-defined mass in the right lumbar region and fullness in the flank region. The laboratory examination results were unremarkable. A plain radiograph of abdomen revealed a large radio-opaque shadow in the right renal region, which was interpreted to be a renal calculus. Ultrasonography revealed an ill-defined hyperechoic mass in the lumbar region, with a central hyperechoic shadowing calculus. In addition, perinephric and parietal wall collections were noted. CT revealed fatty replacement of the right kidney with thin strands of residual, atrophic renal tissue. Small hypodense collections were noted in the perinephric space and extending into the parietal wall. Aspiration revealed pus and foamy cells suggestive of xanthogranulomatous pyelonephritis. There was no laboratory evidence of tuberculosis [Figures 1 and 2]. The patient was treated with nephrectomy and drainage of collections. Pertoperative findings suggested renal replacement lipomatosis with xanthogranulomatous pyelonephritis, which was confirmed by histopathology of the resected tissue.

## Discussion

RRL is characterized by total replacement of kidney by adipose tissue, with varying degrees of fibrotic change. It is considered by some to be a severe form of renal sinus lipomatosis.[2] The possible causative mechanisms include pressure atrophy of renal parenchyma by expanding renal sinus fat, invasion of renal parenchyma by fat and fatty replacement after atrophy of renal parenchyma. It is associated with long-standing inflammation and calculi in 76%-79% of cases.[1,3] The occurrence of RRL in native kidneys following renal transplantation suggests the involvement of other mechanisms as well.[4]

The main differential considerations for RRL include malakoplakia, fat containing tumors such as liposarcomas, lipomas, and angiomyolipomas, xanthogranulomatous pyelonephritis and transitional carcinoma of the renal sinus.[5] RRL and xanthogranulomatous pyelonephritis may occur simultaneously, as seen in this case.[6–8]

Abdominal radiographs reveal calculi, while IVU demonstrates a nonfunctioning or poorly functioning kidney. Ultrasonography shows the replacement of kidney with an echogenic mass with a central calculus; however, the perirenal changes are poorly depicted.[9] CT appears to be the imaging method of choice; the various CT features include marked parenchymal atrophy, abundant adipose tissue in the renal sinus and perirenal regions, and calculi. MRI, with techniques of fat suppression can show information similar to CT. Renal liposarcoma is located peripherally (between the kidney and renal capsule), and does not produce a defect in renal parenchyma. Again, in cases of angiomyolipoma/lipoma, atrophy of renal parenchyma, calculi and absence of renal contrast concentration and excretion are not observed.[5] In cases of xanthogranulomatous pyelonephritis, CT reveals renal calculi, pyonephrosis, infiltration of adjacent soft tissue or abdominal wall, and parenchymal low attenuation densities consistent with abscesses.[10]

The diagnosis of RRL can be elusive with an erroneous initial impression of an angiomyolipoma or liposarcoma. This case is intended to increase the awareness of this very rare entity among radiologists and surgeons.

## References

[1] Yagci C, Kosucu P, Yorubulut M, Akyar S (1999). Renal replacement lipomatosis: Ultrasonography and computed tomography findings. Eur Radiol.

[2] Rha SE, Byun JY, Jung SE, Oh SN, Choi YJ, Lee A (2004). The renal sinus: Pathologic spectrum and multimodality imaging approach. Radiographics.

[3] Karasick S, Wechsler RJ (2000). Replacement lipomatosis of the kidney. Radiology.

[4] Chang SD, Coakley FV, Goldstein RB (2005). Renal replacement lipomatosis associated with renal transplantation. Br J Radiol.

[5] Kocaoglu M, Bozlar U, Sanal HT, Guvenc I (2007). Replacement lipomatosis: CT and MRI findings of a rare renal mass. Br J Radiol.

[6] Sakata Y, Kinoshita N, Kato H, Yamada Y, Sugimura Y (2004). Coexistence of renal replacement lipomatosis with xanthogranulomatous pyelonephritis. Int J Urol.

[7] Prasad KK, Pandey R, Kathuria M, Pradhan PK (2003). Coexistent massive renal replacement lipomatosis and xanthogranulomatous pyelonephritis. Indian J Pathol Microbiol.

[8] Kiris A, Kocacok E, Poyraz AK, Dagli F, Boztosun Y (2005). Xanthogranulomatous pyelonephritis with nephrocutanous fistula and coexisting renal replacement lipomatosis: A report of rare case. Clin Imaging.

[9] Ginat DT, Bhat S, Dogra VS, Guvenc I (2008). Replacement lipomatosis of the kidney: Sonographic features. J Ultrasound Med.

[10] Goldman SM, Hartman DS, Fishman EK, Finizio JP, Gatewood OM, Siegalmann SS (1984). CT of xanthogranulomatous pyelonephritis: Radiologic-pathologic correlation. AJR Am J Roentgenol.

